# Wingless-type MMTV integration site family member 5a: a novel biomarker regulated in type 2 diabetes mellitus and diabetic kidney disease

**DOI:** 10.1007/s40200-019-00461-8

**Published:** 2019-11-22

**Authors:** Wei Xu, Houfa Geng, Xuekui Liu, Xiuli Wang, Rui Li, Qian Lv, Yin Liu, Jie Wang, Manqing Yang, Peter M. Jones, Jun Liang

**Affiliations:** 1 Department of Endocrinology, Xuzhou Central Hospital, Xuzhou Institute of Medical Sciences, Xuzhou Clinical School of Nanjing Medical University, Affiliated Hospital of Medical School of Southeast University, Xuzhou, 221009 Jiangsu China; 2 Department of Diabetes, School of Life Course Sciences, Faculty of Life Sciences and Medicine, King’s College London, London, SE1 1UL UK

**Keywords:** Wingless-type MMTV integration site family member 5a, Type 2 diabetes mellitus, Diabetic kidney disease

## Abstract

**Objectives:**

Type 2 diabetes mellitus (T2DM) is sustained by insulin resistance (IR) and reduced β-cell mass, which is largely due to insulin secretory dysfunction. Wnt5a protein is essential to islet formation and β-cell migration in the development of pancreas in vertebrates. Levels of the Wnt5a protein antagonist plasma secreted frizzled-related protein 5 (Sfrp5) were elevated in patients with T2DM. However, the association between Wnt5a, T2DM patients and diabetic kidney disease (DKD) is unknown. We aim to investigate the circulating Wnt5a levels in in different clinical stages of T2DM and evaluate its correlation of duration of diabetes mellitus chronic complication.

**Methods:**

A total of 329 participants (187 males, 142 females; age range 40 to 80 years) were enrolled in this study. Serum Wnt5a levels were measured by an enzyme-linked immunosorbent assay (ELISA). The demographic and clinical parameters evaluated in subjects with new onset T2DM, onset T2DM after treatment and DKD at different clinical phases.

**Results:**

Wnt5a was significantly down-regulated in newly diagnosed T2DM patients and gradually increased after 3 months of treatment. Interesting, serum wnt5a was gradually increased in patients with long-term diabetes and kidney disease compared to patients with T2DM and onset DKD.

**Conclusions:**

We speculated that the Wnt5a protein might regulate islet function and be involved in the onset of diabetes as a protective factor. It may be one of the inflammatory factors adversely involved in the progression of diabetic nephropathy.

## Introduction

Type 2 diabetes mellitus (T2DM) is one of the most prevalent metabolic disorders in the world, with an estimated 113.9 million adults in China, and we can clearly see that the incidence rate is also dramatically rising with time [1]. The prevalence rate for prediabetes among Chinese adults reported a rate of over 50% [2]. This metabolic disorder not only affects health and quality of life, but can also bring serious physical, psychological and economical burdens to individuals and governments for a variety of chronic complications, such as macrovascular and microvascular complications. Among these complications, renal involvement occurs in a subset of diabetic patients, often playing a key role in morbidity and mortality [3]. Multiple factors have been proposed to cause pathological changes in diabetes mellitus and its renal complications. Previous studies have shown that low-grade chronic inflammation and activation of the renin-angiotensin system (RAS) all accelerate the development of diabetes and its renal complications [4, 5]. However, it is disappointing that the exact pathogenesis leading to T2DM and DKD remain not fully elucidated.

Recently, A lot of evidence has been demonstrated that the contribution of Wnt signalling in both the development and progression of T2DM and DKD. Wnt5a is involved in several non-canonical Wnt signaling pathways, through binding to its receptor Frizzled-5 (Fzd5), acts as a critical regulator in a host of developmental processes and diverse pathogenic situations, such as cancer, fibrosis, and inflammation, and it promotes renal interstitial fibrosis [6–9]. Wnt5a/Fzd5 signalling pathway plays a key role in proper insulin-cell migration and development of pancreatic islet [10]. Previous study demonstrated that levels of the Wnt5a protein antagonist plasma secreted frizzled-related protein 5 (Sfrp5) were elevated in patients with T2DM [11]. Interestingly, our recent in vitro study investigated that high expression of Wnt5a and Fzd5 could be found in islet stellate cells isolated from db/m mice, and wnt5a increased the secretion of insulin after co-culture with β cells [12]. However, the role of Wnt5a protein has not been studied in T2DM and DKD.

In this study, we analysed the expression of wnt5a protein in the presence of T2DM and its chronic complications to determine its association and clinical significance.

## Methods

### Patient recruitment and exclusion criteria

This study was carried out at the endocrinology department of Xuzhou Central Hospital from June 2016 to June 2017 in line with the principles enunciated in the Declaration of Helsinki. This study was reviewed and approved by the Ethical Committees of Xuzhou Central Hospital. Written informed consent was obtained from all of the participants.

A total of 109 consecutive patients with onset T2DM, 110 patients with DKD and 110 normal control patients participated in the study. The diagnosis of T2DM was based on the American Diabetes Association (ADA) 2014 criteria. Patients with onset T2DM had a plasma fasting glucose level > 7.0 mmol/L or 2 h values in the OGTT >11.1 mmol/L) without a previous diabetes diagnosis and were given lifestyle improvement and metformin treatment for 3 months.

Microalbuminuria has been defined as 30 to 300 mg of albumin in a 24-h urine collection, DKD was defined as either the presence of microalbuminuria (30 to 299 mg albumin/24 h or an albumin to creatinine ratio [ACR] of 30 to 299 mg/g) or macroalbuminuria (> 300 mg albumin/24 h or ACR > 300 mg/g). The DKD I/II/III groups were defined as having ACR ≤ 29 mg/g (ACR1 group), 30 to 299 mg/g (ACR2 group) and ≥ 300 mg/g (ACR3 group), respectively.

The participants underwent a routine medical examination. Subjects meeting any of the following criteria were excluded from this study: (1) T1DM; (2) acute complications of diabetes, such as diabetic ketoacidosis, hyperglycaemic hyperosmolar state, lactic acidosis and hypoglycaemic coma; (3) history of other disease known to be associated with nephropathy besides DKD; (4) pregnancy; (5) Patients with heart failure, haematologic disease, liver dysfunction, or taking steroids were also excluded.

### Biochemical assays

Whole blood samples were collected into 5 ml Vacutainer tubes from the control veni-puncture after an overnight fast. In the onset T2DM and DKD groups, the blood samples were drawn on admission before treatment with blood and at the time of discharge from the hospital. The blood samples were drawn again for the onset T2DM group treated by lifestyle improvement and metformin for 3 months. Laboratory tests were performed using laboratories’ routine clinical methods in the hospital laboratory. HbA1c was measured with an HbA1c Meter from Bio-Rad Laboratories, Ltd. (Shanghai, China), with reference value mmol/mol. The concentrations of plasma Wnt5a were detected using the ELISA kit (ISBio, United States of America). The analytical sensitivities were 1.0 ng/mL for Wnt5a.

### Statistical methods

Data are shown as the mean ± standard deviation (SD). SAS statistical software (version 9.1; SAS Institute, Inc.) was used for data management and statistical analyses. Analysis of variance (ANOVA) statistical technique is conducted to examine differences between groups. Correlations of Wnt5a and clinical parameters were performed using Pearson’s correlations or Spearman’s rank correlation coefficient. The odds ratio (OR) of Wnt5a for DKD was determined by logistic regression models adjusted for covariates, including age, BMI, SBP, DBP, FBG, TG, TC, HDL and LDL. All reported *P*-values are two-tailed. Variables with P-values <0.05 were considered statistically significant.

## Results

### Participants and baseline characteristics

A total of 329 subjects (187 males, 142 females; age range 40–80 years) participated in the study. The baseline demographic and clinical characteristics of the three enrolled populations are summarized in Table 1. The glycaemic variables were significantly higher in the two diabetic patient groups (plasma glucose: 7.28 ± 2.64 mmol/L and 8.04 ± 3.07 mmol/L, respectively; HbA1c: 7.84 ± 3.37% and 8.3 ± 4.23%, respectively, for onset T2DM and DKD) than in the control group (plasma glucose: 5.14 ± 1.32 mmol/L, HbA1c: 5.21 ± 1.87%) (all *P* < 0.001). With respect to age and gender, there were no significant differences among the three groups. The BMIs were significantly higher in the two diabetic patient groups (27.9 ± 9.28 and 25.4 ± 4.03 for onset T2DM and DKD, respectively) than in the control group (22.1 ± 3.54). Both the systolic and diastolic blood pressures were lower in the control group and higher in the onset T2DM and DKD group (*P* < 0.001).

**Table 1:** The clinical characteristics of subjects in the three groups

	Control	Onset T2DM	T2DM with DKD	F/t	P
N	110	109	110		
Female	45	47	50		
Age (years)	54.3 ± 12.42	59.8 ± 10.51	63.70 ± 11.55	18.48	<0.001
Duration (months)		5.01 ± 3.04	113.6 ± 49.61	22.91	<0.001
BMI (kg/m^2^)	22.1 ± 3.54	27.9 ± 9.28	25.40 ± 4.03	24.30	<0.001
SBP (mmHg)	112.8 ± 14.72	118.8 ± 16.34	136.9 ± 19.30	60.63	<0.001
DBP (mmHg)	71.40 ± 9.86	72.40 ± 6.87	83.70 ± 9.14	67.40	<0.001
TC (mmol/L)	4.12 ± 0.84	4.60 ± 1.02	5.01 ± 1.24	19.94	<0.001
TG (mmol/L)	1.13 ± 0.62	1.64 ± 1.24	2.00 ± 1.33	17.10	<0.001
HDL (mmol/L)	1.32 ± 0.33	1.14 ± 0.28	1.08 ± 0.19	23.04	<0.001
LDL (mmol/L)	2.24 ± 0.54	2.78 ± 1.31	3.01 ± 1.87	9.360	<0.001
FBG (mmol/L)	5.14 ± 1.32	7.28 ± 2.64	8.04 ± 3.07	41.14	<0.001
2hPG (mmol/L)	6.13 ± 2.71	11.37 ± 3.68	11.18 ± 4.08	77.55	<0.001
HbA1c (%)	5.21 ± 1.87	7.84 ± 3.37	8.30 ± 4.23	27.99	<0.001

Clinical characteristics of different groups of subjects. Data are expressed as the means ± SD or as percentages for normal distribution. Non-normally distributed values are presented as medians (IQR)

*BMI* Body mass index, *SBP* Systolic blood pressure, *DBP* Diastolic blood pressure, *TC* Total cholesterol, *TG* Triglyceride, *HDL* High-density lipoprotein, *LDL* Low-density lipoprotein, *FPG* Fasting plasma glucose, *2hPG* 2-h plasma glucose, *HbA1c* haemoglobin A1c

### Wnt5a levels decreased in onset T2DM and gradually increased after treatment

**Fig. 1:**
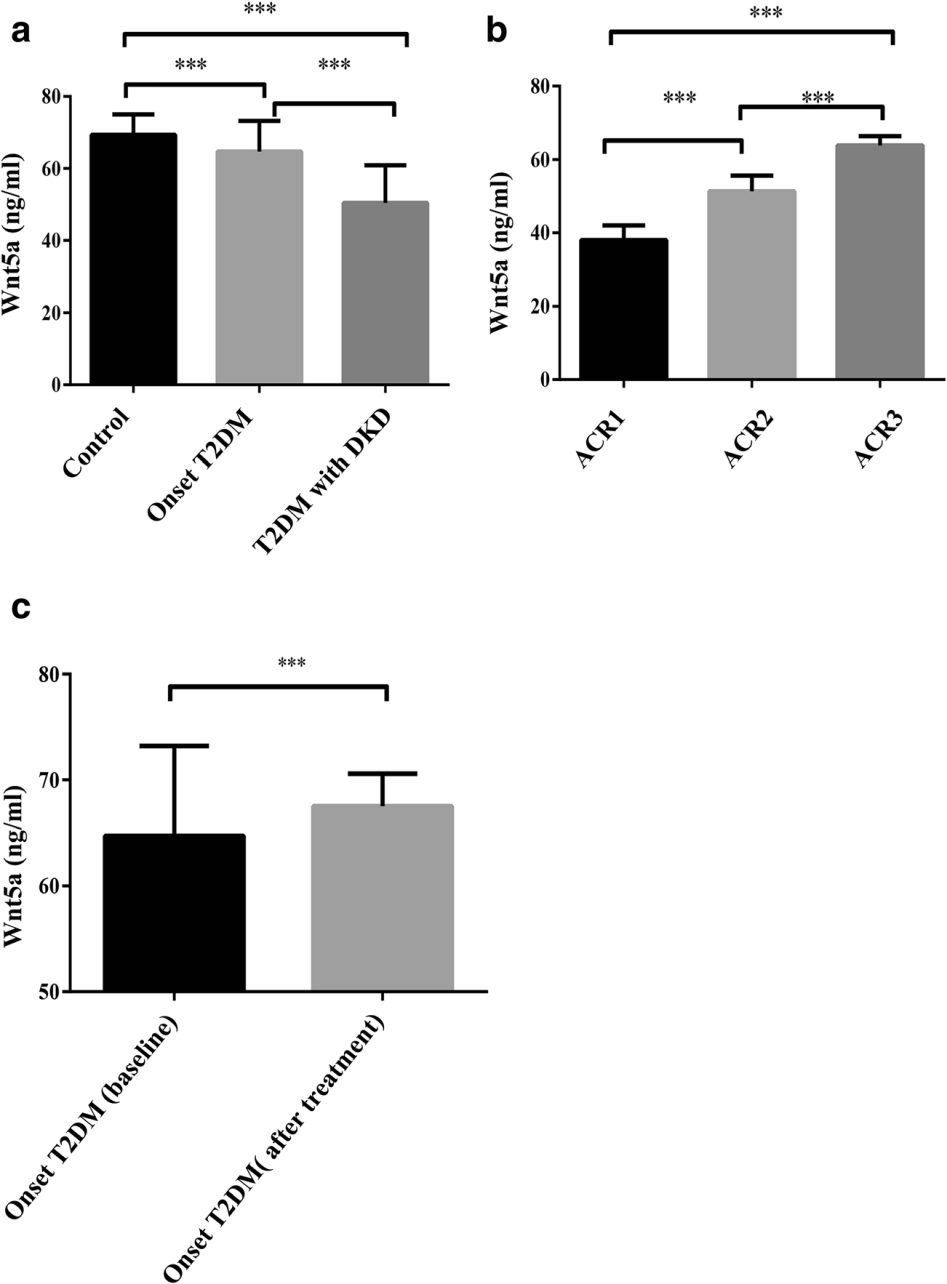
Serum levels of Wnt5a in different groups of the study population. **a** Wnt5a serum levels decreased significantly in newly diagnosed (64.73 ± 8.49 ng/ml, *p* < 0.001) and T2DM with DKD patients (50.42 ± 10.46 ng/ml, p < 0.001) compared to healthy controls (69.38 ± 5.68 ng/ml). Data are presented as the means ± SD, *p < 0.05, **p < 0.001. **b** Wnt5a serum levels increased slightly in DKDII (ACR ≥ 30, ACR ≤ 299) (51.34 ± 4.30 ng/ml, p < 0.001) and DKDIII (ACR ≥ 300) (63.89 ± 2.57 ng/ml, p < 0.001) compared to DKDI (38.02 ± 4.02 ng/ml). Data are presented as the means ± SD, *p < 0.05, **p < 0.001. **c** Wnt5a serum levels increased significantly after treatment with lifestyle improvement and metformin for 3 months (67.52 ± 3.06 ng/ml, *P* < 0.001, p < 0.001) compared to onset T2DM patients (64.73 ± 8.49 ng/ml). Data are presented as the means ± SD, *p < 0.05, **p < 0.001

We measured fasting serum Wnt5a levels in the subjects with onset T2DM and onset T2DM after treatment using a newly developed ELISA assay (Table 2 and Fig. 1a, c). Wnt5a levels decreased in the diabetic group patients compared to healthy controls (64.73 ± 8.49 ng/ml versus 69.38 ± 5.68 ng/ml, *P* < 0.001), and increased Wnt5a was detected after 3 months of treatment (64.73 ± 8.49 ng/ml versus 67.52 ± 3.06 ng/ml, P < 0.001).

**Table 2:** The clinical characteristics of onset T2DM group before and after treatment

	Onset T2DM (baseline)	Onset T2DM (after treatment)	t	*P*
n	109	104		
Wnt5a (ng/ml)	64.73 ± 8.49	67.52 ± 3.06	3.228	0.001
BMI (kg/m^2^)	27.9 ± 9.28	25.14 ± 2.41	3.008	0.003
SBP (mmHg)	118.8 ± 16.34	104.8 ± 16.47	6.225	<0.001
DBP (mmHg)	72.40 ± 6.87	81.05 ± 10.94	−6.873	<0.001
TC (mmol/L)	4.60 ± 1.02	4.03 ± 0.75	4.661	<0.001
TG (mmol/L)	1.64 ± 1.24	1.15 ± 0.53	3.779	<0.001
HDL (mmol/L)	1.14 ± 0.28	1.54 ± 0.87	−4.472	<0.001
LDL (mmol/L)	2.78 ± 1.31	2.05 ± 0.83	4.881	<0.001
FBG (mmol/L)	7.28 ± 2.64	6.07 ± 1.44	4.178	<0.001
2hPG (mmol/L)	11.37 ± 3.68	8.21 ± 1.98	7.853	<0.001
HbA1c (%)	7.84 ± 3.37	6.57 ± 2.04	3.344	0.001

Clinical characteristics of onset T2DM group and lifestyle improvement and metformin treatment for 3 months. Data are expressed as mean ± SD or as percentages for normal distribution. Non-normally distributed values are presented as median (IQR)

*BMI* Body mass index, *SBP* Systolic blood pressure, *DBP* Diastolic blood pressure, *TC* Total cholesterol, *TG* Triglyceride, *HDL* High-density lipoprotein, *LDL* Low-density lipoprotein, *FPG* Fasting plasma glucose, *2hPG* 2-h plasma glucose, *HbA1c* Haemoglobin A1c

### Wnt5a levels changed with the duration of DKD

**Fig. 2:**
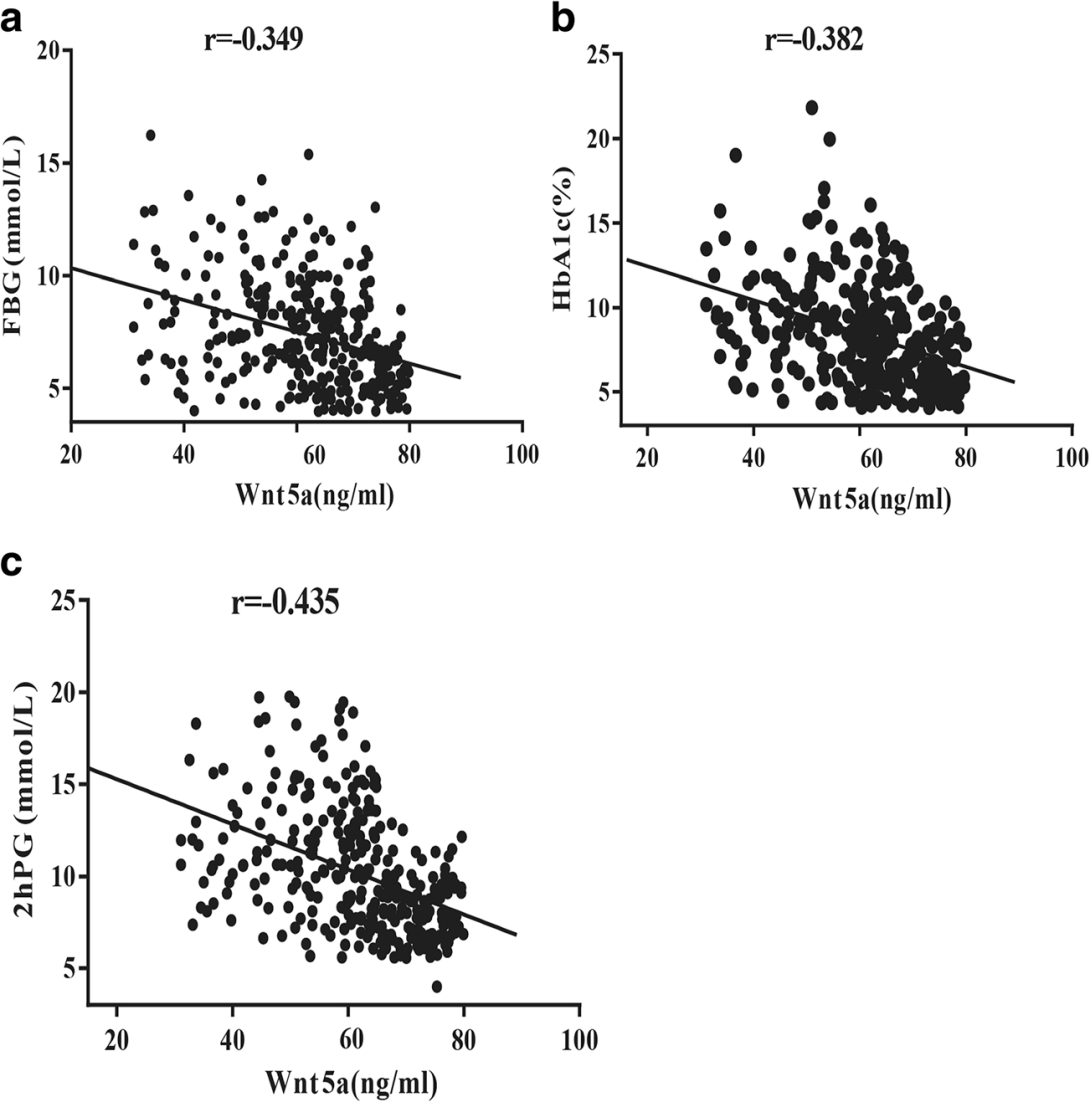
Correlation analysis of Wnt5a in T2DM patients. **a** Correlation of Wnt5a with FBG (Spearman r = −0.349, p < 0.001). **b** Correlation of Wnt5a with HbA1c (Spearman r = −0.435, p < 0.001). **c** Correlation of Wnt5a with 2hPG (Spearman r = −0.382, p < 0.001)

Wnt5a levels were clearly decreased in DKD patients compared to onset T2DM and healthy controls (50.42 ± 10.46 ng/ml versus 69.38 ± 5.68 ng/ml and 64.73 ± 8.49 ng/ml, P < 0.001) (Fig. 1a and Table 3). A negative correlation between FBG, 2hPG, HbA1c and Wnt5a was also found in all the participants (r = −0.349, −0.435, −0.382, respectively) (Fig. 2a, b, c). However, there was no association between Wnt5a and age, gender, BMI and the systolic and diastolic blood pressures.

**Table 3:** Serum levels of Wnt5a in different groups

GROUP		n	Wnt5a ng/ml	*F*^*a*^	*P*^*a*^	*F*^*b*^	*P*^*b*^
Control		110	69.38 ± 5.68	150	<0.001		
Onset T2DM (baseline)		109	64.73 ± 8.49				
DKD		110	50.42 ± 10.46				
DKD I	ACR ≤ 29	34	38.02 ± 4.02			350.6	<0.002
DKD II	ACR ≥ 30, ACR ≤ 299	48	51.34 ± 4.30				
DKD III	ACR ≥ 300	28	63.89 ± 2.57				

Analysis of control group, onset T2DM group(baseline) and T2DM with DKD group by one-way analysis of variance; Fb and Pb: Analysis of different groups form levels of microalbuminuria by one-way analysis of variance

*DKD* Diabetic kidney disease, *ACR*, An albumin to creatinine ratio

Notably, we found that Wnt5a levels were gradually higher in the DKD II/III group compared with those in DKD I group (51.34 ± 4.30 ng/ml and 63.89 ± 2.57 ng/ml versus 38.02 ± 4.02 ng/ml, *P* < 0.001) (Fig. 1b). There was a positive correlation between Wnt5a and ACR. In the logistic regression model adjusted for age and BMI, the odds ratio [OR, 95% confidence interval (95%CI)] was 1.12 (1.081~1.177). After further adjustment for SBP, DBP and FBG, the odds ratio was 1.321 (1.21~1.492). In the three models adjusted for age, BMI, SBP, DBP, FBG, TG, TC, HDL and LDL, the odds ratio was 1.422 (1.271~1.658) (Table 4).

**Table 4:** The association between Serum levels of Wnt5a and end-stage renal

	B	world	*P*	*OR*	95%CI
Model1	2.123	113.98	<0.001	1.12	1.081~1.177
Model2	1.135	27.254	<0.001	1.321	1.21~1.492
Model3	0.863	14.477	<0.001	1.422	1.271~1.658

Model 1: adjusted for age and BMI

Model 2: adjusted for age, BMI, SBP, DBP, FBG

Model 3: adjusted for age, BMI, SBP, DBP, FBG, TG, TC, HDL, LDL

In those three models, we had added a variable which transformed by the levels of ACR in the DKD group. The variable named end-stage renal. When the level of ACR was equal or greater than 300 mg/g, we had marked the end-stage renal as 1, others as 0. This above results of multivariate logistic regression analysis are presented as the OR of end-stage renal and Wnt5a levels

## Discussion

The present study analysed wnt5a levels in patients with different stages of T2DM. First, patients with early onset T2DM and DKD had significantly decreased wnt5a levels. However, our study also provided interesting evidence that wnt5a gradually increased in long-term T2DM patients with kidney disease. To our knowledge, this is the first report to describe the expression of wnt5a protein playing difference roles in the presence of T2DM and its chronic kidney complications.

In the present study, we have shown that Wnt5a gradually decreased in patients with onset diabetes and gradually increased after treatment with lifestyle improvement and metformin for 3 months, albeit at a relatively high level under normal conditions, which may provide some insight into one mechanism by which Wnt5a protein may influence development of T2DM. The causes of type 2 diabetes include both genetic, lifestyle and environmental elements that affect β-cell function and insulin sensitivity [13]. However, the mechanisms controlling the interplay of these three impairments remain not fully elucidated. Previous studies have shown that the deposition of islet amyloid and low-grade chronic inflammation all associated with the procession of T2DM [14]. Numerous pro-inflammatory cytokines such as IL-1b, TNF-a, and IL-6 is the most promising part for development of T2DM. These cytokines released from adipose tissues induce inflammation not only in the corresponding tissue but also in the β-cells of pancreatic islets and block the activation of insulin signaling receptors, ultimately linked to insulin resistance [15–17].

Wnt5a is involved in several non-canonical Wnt signaling pathways, and it is a prototypical wnt of the β-catenin-independent branch, which is highly conserved among species and plays key roles in numerous biological processes. Wnt5a signalling is associated with several human pathologies such as pulmonary fibrosis, renal fibrosis, post-infarct cardiac remodelling and hypertrophic scars [18–22]. Several studies suggested that wnt5a is also expressed in mammalian adipose tissue and expression in mice was induced by a high-fat diet and obesity [23]. Wnt5a/Fzd5 signalling plays a role in proper insulin-cell migration and islet formation during vertebrate pancreatic development [10]. Plasma levels of the Wnt5a protein receptor Sfrp5 were elevated in patients with T2DM [11]. However, the underlying mechanisms of Wnt5a-β cells in diabetes remain to be elucidated. Interestingly, our recent in vitro study showed that Wnt5a protein expression from islet stellate cells (ISCs) was higher in db/m mice than in db/db mice, and Wnt5a increased the secretion of insulin by co-culture with islets; the results revealed that the Wnt5a protein is a key modulator of ISC-mediated maintenance of islet function [12].

In the current study, we found negative correlations between wnt5a levels and HbA1c, as well as fasting blood glucose (FBG) and 2-h postprandial glucose (2hPG), implying that the decreased wnt5a levels might have a close association with insulin resistance and β cell dysfunction. The decreased wnt5a level observed in patients with onset T2DM in this study may suggest that changes in the wnt5a level represent a regulatory mechanism to counteract metabolic stress and β cell dysfunction in diabetes. The results revealed that Wnt5a protein maybe a key modulator in the development of T2DM.

Our studies have identified that patients with CKD have significantly decreased Wnt5a levels. However, our studies have also revealed that Wnt5a levels were gradually higher in the DKD II/III group than in the DKD I group. Diabetic kidney disease (DKD) is one of the most frequent and dangerous microvascular complications in diabetes mellitus and is independently correlated with an increased risk of developing end stage renal failure (ESRD) [24–26]. Multiple factors have been proposed to cause pathological changes in T2DM patients with kidney disease, such as the interactions between hyperglycaemia-induced hemodynamic and metabolic disorders, as well as the accumulation of extracellular matrix (ECM) proteins in the kidney microenvironment [27]. However, the exact pathogenesis controlling the DKD remain not fully elucidated. Wnt/β-catenin signaling pathway is re-activated in a wide variety of animal models of proteinuric kidney and in human kidney disorders [28–31]. Whereas some study demonstrated to a protective role of Wnt signalling in repair after acute kidney injury, Emerging evidence suggests that sustained activation of Wnt/β-catenin is associated with the development and progression of renal fibrosis [32–36]. However, the Wnt/β-catenin mechanisms controlling interplay of DKD are unclear. In the present study, we analysed wnt5a levels in patients with DKD and revealed a close relationship between wnt5a levels and DKD patients. Serum wnt5a was gradually increased in long-term diabetes patients with kidney disease compared to T2DM with onset DKD patients. More importantly, these results further imply that wnt5a signalling may play an important role in facilitating the development and progression of DKD. However, this needs to be clarified in future studies. The mechanisms underlying diabetes and its microvascular chronic complications are multiple and complex, and our study population was relatively small. Thus, Further studies with larger populations are required to elucidate fully the role of Wnt5a in patients with different stages of T2DM. We speculate that the Wnt5a protein may regulate islet function and be involved in the onset of diabetes as protective factors. It may be one of the inflammatory factors adversely involved in the progression of diabetic nephropathy.

Some limitations of this study need to be considered. First, owing to its observational characteristic, this hypothesis requires a randomized, controlled trial to definitively determine the role of Wnt5a in patients with T2DM. Finally, the exact origin of decreased Wnt5a in onset T2DM and gradually increased trend in DKD patients is unknown. Thus, further studies with larger populations and basic research are required.

In conclusion, we report that Wnt5a levels correlated with the duration of diabetes. Therefore, Wnt5a might play an important role in the development of type 2 diabetes mellitus and its renal complications. Future trials are required to reassess performance and evaluate the mechanisms of Wnt5a-β cell-microvascular complications.
